# Increased gene expression of *FOXP1* in patients with autism spectrum disorders

**DOI:** 10.1186/2040-2392-4-23

**Published:** 2013-07-01

**Authors:** Wei-Hsien Chien, Susan Shur-Fen Gau, Chun-Houh Chen, Wen-Che Tsai, Yu-Yu Wu, Po-Hsu Chen, Chi-Yung Shang, Chia-Hsiang Chen

**Affiliations:** 1 Department of Occupational Therapy, College of Medicine, Fu-Jen Catholic University, No. 510, Zhongzheng Rd, New Taipei City, Taiwan; 2 Department of Psychiatry, National Taiwan University College of Medicine, No.1 Jen-Ai Rd. Section 1, Taipei, Taiwan; 3 Department of Psychiatry, National Taiwan University Hospital, No. 7, Chung-Shan South Rd, Taipei, Taiwan; 4 Department of Psychology and School of Occupational Therapy, Graduate Institute of Brain and Mind Sciences, Graduate Institute of Epidemiology and Preventive Medicine, National Taiwan University, Taipei, Taiwan; 5 Institute of Statistical Science, Academia Sinica, No. 128, Academia Rd. Nankang, Taipei, Taiwan; 6 Department of Psychiatry, Chang Gung Memorial Hospital at Linkou, 5 Fusing Street, Kueishan, Taoyuan, Taiwan; 7 Department of Statistics, Ohio State University, 1958 Neil Ave, Columbus, OH, USA

**Keywords:** Autism, FOXP1, Expression microarray, Genetics, Lymphoblastoid cell line

## Abstract

**Background:**

Comparative gene expression profiling analysis is useful in discovering differentially expressed genes associated with various diseases, including mental disorders. Autism spectrum disorders (ASD) are a group of complex childhood-onset neurodevelopmental and genetic disorders characterized by deficits in language development and verbal communication, impaired reciprocal social interaction, and the presence of repetitive behaviors or restricted interests. The study aimed to identify novel genes associated with the pathogenesis of ASD.

**Methods:**

We conducted comparative total gene expression profiling analysis of lymphoblastoid cell lines (LCL) between 16 male patients with ASD and 16 male control subjects to screen differentially expressed genes associated with ASD. We verified one of the differentially expressed genes, *FOXP1*, using real-time quantitative PCR (RT-qPCR) in a sample of 83 male patients and 83 male controls that included the initial 16 male patients and male controls, respectively.

**Results:**

A total of 252 differentially expressed probe sets representing 202 genes were detected between the two groups, including 89 up- and 113 downregulated genes in the ASD group. RT-qPCR verified significant elevation of the *FOXP1* gene transcript of LCL in a sample of 83 male patients (10.46 ± 11.34) compared with 83 male controls (5.17 ± 8.20, *P* = 0.001).

**Conclusions:**

Comparative gene expression profiling analysis of LCL is useful in discovering novel genetic markers associated with ASD. Elevated gene expression of *FOXP1* might contribute to the pathogenesis of ASD.

**Clinical trial registration:**

Identifier: NCT00494754

## Background

Autism spectrum disorders (ASD) represent a constellation of childhood-onset neurodevelopmental disorders characterized by three core symptom domains: deficits in reciprocal social interactions, impairments in language development and verbal communication, and the presence of stereotyped, repetitive behaviors and restricted interests [1]. The clinical presentations of ASD are highly heterogeneous with varied expressivity and differences in the severity of clinical symptoms and functional impairments. The estimated prevalence of ASD in the general population worldwide is approximately 1 to 2.6%, with a male predominance [2,3]. The etiology of ASD is mainly attributable to genetic factors. The estimated heritability of ASD is more than 90%, and the genetic underpinnings of ASD are heterogeneous and complex, involving multiple genes, gene-gene interactions, and gene-environmental interactions [4,5]. Identification of genetic underpinnings can shed light on the pathogenesis of ASD, and facilitate the development of new treatments for ASD. The genetic risk factors for ASD identified so far range from common variants conferring a small clinical effect to rare mutations that confer a high clinical effect [6]. The high heterogeneity of ASD may account for the varied clinical presentations of patients with ASD. Several genes have been found to be associated with ASD, but more genes remain to be discovered.

Microarray-based gene expression profiling is a useful technology allowing simultaneous measurement of hundreds to thousands of gene transcripts, and is useful for large-scale gene discovery [7]. This technology has been used in several post-mortem brain studies of psychiatric disorders, including schizophrenia, bipolar disorder, and autism [8-11]. Since there are several limitations to the use of the post-mortem brain tissue in gene expression studies, and the study of fresh brain tissue from living psychiatric patients is impractical at the present time, several studies have reported the use of peripheral blood cells and lymphoblastoid cell lines (LCL) as surrogates for brain tissue in the gene expression studies of mental disorders [12-16]. In addition, a moderate correlation of gene expression between peripheral blood cells and brain tissue in humans has been reported, supporting the usefulness of peripheral blood cells in the gene expression studies for psychiatric research [17].

Comparative gene expression profiling analysis of LCL has been conducted in several autism studies [18-22]. For example, several differentially expressed genes important to the development, structure, and/or function of the nervous system were detected in monozygotic twins discordant for autism using this method [18]. Another genome-wide expression profiling of LCL identified shared pathways among different forms of autism [19]. Also, altered pathways in neural development and steroid biogenesis were detected in a study of total gene expression profiling analysis of LCL in autistic patients and their unaffected sib-pairs [20]. Another study found that the imbalance of cellular and mitochondrial glutathione redox in LCL was associated with autism [21]. LCL was also used successfully to investigate the role of microRNA in autism [22]. Taken together, these studies support the usefulness of comparative gene expression profiling analysis of LCL in the molecular genetic study of autism [23].

The above-mentioned findings attracted our interest in identifying novel differentially expressed genes associated with autism in our population using comparative gene expression profiling analysis of LCL. We first conducted a comparative gene expression profiling analysis of LCL in a sample of 16 male patients with ASD and 16 male control subjects as an initial screening of differentially expressed genes associated with autism. In the second stage, we verified the authenticity of a selected differentially expressed gene, *FOXP1*, in an expanded sample of 83 male patients and 83 male control subjects using real-time quantitative PCR (RT-qPCR).

## Methods

### Subjects

The study was conducted in two tiers. In the first experiment, microarray-based total gene expression profiling analysis was performed with 20 male patients with ASD and 20 male control subjects, but only 16 of each group passed the quality control of the experiment and they were subjected to further analysis. In the second experiment, the transcript level of *FOXP1* was compared between 83 male patients with ASD and 83 healthy male controls. The sample included the 20 male patients and 20 male control subjects from the first tier of the experiment. All the patients were recruited from the outpatient clinics of psychiatric departments in two medical centers (National Taiwan University Hospital [NTUH] in Taipei and Chang-Gung Memorial Hospital (CGMH in Kueishan). All subjects were Han Chinese. The clinical diagnoses of probands were made by senior board-certified child psychiatrists according to the DSM-IV diagnostic criteria for autistic disorder or Asperger’s disorder, and were further confirmed by structural interviews using the Chinese version of the Autism Diagnostic Interview-Revised (ADI-R) [24-27]. The ADI-R [24] is a standardized, comprehensive, semi-structured, investigator-based interview scale that was designed for interviewing the caregivers/parents to obtain information on the developmental and behavioral aspects of ASD of children with a mental age from about 18 months to adulthood. The behavioral aspects of ASD assessed by the ADI-R include reciprocal social interactions, communication, and repetitive stereotyped behaviors/interests. Gau and colleagues have prepared the Chinese version of the ADI-R, which was approved by the World Psychological Association (WPS) in May 2007, for use in this study [25-27].

The case group in the first tier of the experiment consisted of 20 male probands, aged 9.1 ± 3.2 years (range 4 to 18 years old). These participants were selected from a family cohort of 393 probands with ASD (373 with autistic disorder and 20 with Asperger’s disorder) [27] because they had the highest severity scores for problems in verbal communication and social reciprocity based on the ADI-R interview and clinical assessments. Probands in the original cohort with fragile X syndrome or Rett’s disorder, and probands who had a previously identified chromosomal abnormality associated with autism, or had any other neurological or medical conditions, were excluded from the study. The control group consisted of 20 males (mean age: 37.15 ± 16.90 years; range 18 to 67 years) who were recruited among the cohort of individuals receiving regular medical checkups in a local medical center.

In the second tier of the experiment, a total of 83 male patients with ASD (including 20 from the first tier of the experiment), aged 10.2 ± 4.6 years (range 3 to 23 years), were recruited. The control group consisted of 83 males (including 20 from the first tier of the experiment), aged 43.5 ± 12.7 years (range 18 to 63 years) who were recruited from among individuals who received regular medical checkups in a local medical center. The mental status and history of mental illness of each control subject were evaluated by a senior psychiatrist using the Mini International Neuropsychiatric Interview (MINI); subjects diagnosed with a DSM-IV axis 1 disorder including ASD were excluded. The Research Ethics Committee of the two research sites approved this study (IRB ID: 9561709027 for NTUH and C20060905 for CGMH). Written informed consent and child assent were obtained from the parents and the probands, respectively, after the purposes and procedures of the study were fully explained and confidentiality was ensured.

### Preparation of lymphoblastoid cell line, total RNA and cDNA

Immortalized LCL from each subject was established by transforming lymphocytes with Epstein-Barr virus (EBV) following the procedures described elsewhere [16]. Total RNA from the LCL was extracted using TRIZOL Total RNA Isolation Reagent according to the manufacturer’s instructions (Invitrogen Life Technologies, Carlsbad, California, USA). The cDNA was prepared using Superscript II RNase H^-^ Reverse Transcriptase (Invitrogen Life Technologies). The details were described in our previous report [16].

### Microarray-based total gene expression experiment

The microarray experiment was performed at the Core Laboratory of National Taiwan University Hospital, Taipei Taiwan using the Affymetrix Human Genome U133 Plus 2.0 Array (Affymetrix Inc., Santa Clara, CA, USA). The experimental procedures followed the protocols provided by the manufacturer.

### Identification of differentially expressed genes

The intensity files of all the subjects were input into the computer program GAP: Generalized Association Plots [28,29] (http://gap.stat.sinica.edu.tw/Software/GAP/index.htm) for quality control using visualization and descriptive statistics. The chips that passed the quality control were subjected to further differentially expressed gene analysis. The data of the chips were normalized using the Robust Multi-array Analysis (RMA) method [30]. In order to filter out probe sets with low variations and to reduce the impact of multiple comparisons, we kept only the 1,000 probe sets with the largest standard deviations. A differentially expressed probe set was defined as a *P* value smaller than 0.05 for a Student’s *t*-test adjusted by the false discovery rate [31] of 5% and fold-change greater than 2.

### Pathway analysis

We performed gene set enrichment analysis (GSEA) on the entire expression profile for all 32 gene chips using the GSEA software (v2.0.10) (http://www.broadinstitute.org/gsea/index.jsp) [32,33]. The phenotype of subject category (ASD and Control) was employed as phenotype annotation for GSEA.

In addition to GSEA, we also carried out pathway analysis using DAVID (Database for Annotation, Visualization and Integrated Discovery) [34] and verified the results from GSEA (v2.0.10) and DAVID (v6.7) with hypergeometric test using the runHyperKEGG procedure in EMA v1.3.1 (A R package for Easy Microarray data Analysis) [35]. Analysis with all three environments (GSEA, DAVID, and EMA) was based on the gene population of Affymetrix Human Genome U133 Plus 2.0 Array annotation data (chip hgu133plus2) with KEGG v3.1 gene symbol as the gene set and pathway database. The lists of up- and downregulated gene probe sets were fed to DAVID and runHyperKEGG.

### Real-time quantitative PCR

RT-qPCR was performed using the SYBR Green method and implemented in the StepOnePlus Real-Time PCR System according to the manufacturer’s protocol (Applied Biosystems, Forster City, CA, USA). The detailed procedures were described in our previous report [16]. The relative standard curve method was used for quantification of mRNA expression (User Bulletin #2 ABI PRISM 7700 sequence detection system). With this method, serial dilutions of known amounts of RNA from a reference sample (pooled from 40 LCLs of male controls) were used to generate an external standard curve. For each unknown sample the relative amount was calculated using linear regression analysis from their respective standard curves. The expression level of *FOXP1* mRNA was normalized by 18S rRNA. The18S rRNA reference gene was measured using pre-developed TaqMan assay reagents 18S rRNA MGB according to the manufacturer’s protocol (Applied Biosystems). All experiments were performed in duplicate. Primer sequences for PCR amplification were designed using online Primer3 software (http://bioinfo.ut.ee/primer3/). All the sequences of primer sets used for RT-qPCR are listed in Table 1.

**Table 1:** Sequences of primers used in the real-time quantitative PCR experiments in this study

**Gene**	**Forward primer**	**Backward primer**	**Ta**
*FOXP1*	5′-CAGATATTGCGCAGAACCAA-3′	5′-GCAAACATTCGTGTGAACCA-3′	60
*FOXP2*	5′-AGCAAATGCAGCAGATCCTT-3′	5′-TGCTGCAAAAGCTGAAGATG-3′	60
*CNTNAP2*	5′-CAGCTCCTCCTCCATCTCTG-3′	5′-TCACCCAATCTGAGCTGCTA-3′	60

Ta, annealing temperature. *FOXP1*[GenBank:NM_001012505.1], *FOXP2*[GenBank:NM_148898.3], *CNTNAP2*[GenBank:NM_014141.5].

## Results

### Characteristics of patients with Autism Spectrum Disorders

The ADI-R interviews regarding the past behaviors revealed that the 16 patients, who underwent the microarray experiment, scored 24.31 ± 3.88 (range, 14 to 28) in the ‘qualitative abnormalities in reciprocal social interaction’ (cut-off = 10), 17.69 ± 2.98 (range, 14 to 28) in the ‘qualitative abnormalities in communication, verbal’ (cut-off = 8), 8.94 ± 2.72 (range, 4 to 14) in the ‘qualitative abnormalities in communication, nonverbal’ (cut-off = 7), and 8.69 ± 2.24 (range, 5 to 12) in the ‘restricted, repetitive and stereotyped patterns of behaviors’ (cutoff = 3). The average age at which the 16 patients said their first word was 27.5 ± 9.9 (range, 12 to 42) months and at which they said their first phrase was 47.5 ± 12.4 (range, 30 to 78) months. Their current average intelligence quotients (IQ) were 82.13 ± 16.23 (range 62 to 117) for full-scale IQ, 89.13 ± 14.63 (range, 68 to 113) for performance IQ, and 78.44 ± 20.34 (range, 45 to 118) for verbal IQ assessed by the Wechsler Intelligence Scale for Children-3rd edition.

The ADI-R results from the parents of 83 patients showed that the 83 patients scored 24.07 ± 4.78 in the ‘qualitative abnormalities in reciprocal social interaction’ (cut-off = 10), 18.05 ± 3.74 in the ‘qualitative abnormalities in communication, verbal’ (cut-off = 8), 10.14 ± 2.41in the ‘qualitative abnormalities in communication, nonverbal’ (cut-off = 7), and 7.76 ± 2.41 in the ‘restricted, repetitive and stereotyped patterns of behaviors’ (cutoff = 3). The average age at which first word was said was 33.3 ± 16.6 (range, 12 to 84) months and that they had their first phrase was said was 49.9 ± 23.1 (range, 15 to 138) months. The average full-scale IQ, performance IQ, and verbal IQ of these 83 patients were 89.31 ± 21.52 (range, 62 to 130), 93.74 ± 19.61 (range, 68 to 140), and 86.89 ± 24.49 (range, 46 to 125), respectively, as assessed by the Wechsler Intelligence Scale for Children-3rd edition. All the probands had abnormal development evident before 30 months of age based on the ADI-R interview and validated by the medical records.

### Identification of differentially expressed genes

The microarray data of 16 patients and 16 male controls passed the quality control. The data of 32 gene chips were then normalized using the RMA method [30]. The descriptive statistics for the distribution of standard deviations of all 54,675 probe sets were in the ranged from 0.025 to 3.668 with the 25th percentile as 0.157, median as 0.202, the 75th percentile as 0.298, and mean as 0.260; and the 98.17th (1,000 among 54,675) percentile was 0.835. Two hundred and fifty-two probe sets of 1,000 probe sets had standard deviations greater than 0.835 expressed differentially among the two groups with a *P* value less than 0.05 for a Student’s *t*-test adjusted by the false discovery rate [31] of 5% and fold-change greater than 2. These 252 differentially expressed probe sets corresponded to 202 genes with 89 of them as upregulated and 113 as downregulated transcripts in the ASD group compared to the control group. The list of differentially expressed genes is shown in Additional file 1.

### Pathway analysis

We performed gene set enrichment analysis on the entire expression profile of all 32 gene chips using the GSEA software (v2.0.10). The most significant 20 KEGG signaling pathways for both up- and downregulated gene sets in the significance order (size of *P* values) of GSEA are listed in the Additional file 2. The results of pathway analysis of DAVID and runHyperKEGG are also shown in the Additional file 2. Seven pathways were commonly found in three analyses, including the long-term depression, the vascular smooth muscle contraction, the arrhythmogenic right ventricular cardiomyopathy (ARVC), the glycerophospholipid metabolism, the allograft rejection, the Jak-STAT signaling pathway, the toll-like receptor signaling pathway, and the RIG-I-like receptor signaling pathways.

### Verification by real-time quantitative PCR

**Figure 1:**
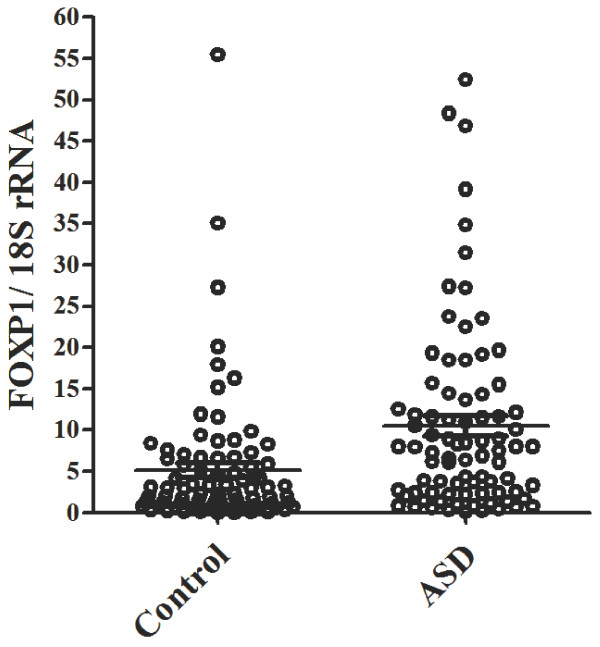
**Scatter plot of the *FOXP1 *mRNA level as normalized by 18S rRNA in 83 male patients with Autism Spectrum Disorders (ASD) and 83 male control subjects.** Horizontal line indicates the mean of *FOXP1* transcript.

The authenticity of the differential expression of *FOXP1* was first verified between the 20 ASD and 20 control subjects using RT-qPCR. The average expression level of *FOXP1* in the 20 patients with ASD (4.57 ± 3.04) was significantly higher than that of the control subjects (1.89 ± 2.64, *P* = 0.005). The upregulation of *FOXP1* gene expression was further supported by an expanded sample size of 83 male ASD patients and 83 male control subjects. This expanded sample size included the initial 20 patients and 20 control subjects. The average transcript level of *FOXP1* in the patient group (10.46 ± 11.34) was significantly higher than that in the control group (5.17 ± 4.20, *P* = 0.001). The distribution and mean of the *FOXP1* transcript level of LCL in 83 patients and 83 controls are shown in Figure 1.

## Discussion

Aberrant language development is one of the clinical characteristics of autism. Mutations of *FOXP2* have been linked to speech and language disorders and ASD [36-38]. FOXP1 is a member of subfamily P of the forkhead box (FOX) transcription factor family and forms a heterodimer with FOXP2 [39,40]. The elevated mRNA level of *FOXP1* in patients with ASD in the initial expression microarray experiment attracted our attention; hence, it was selected for further validation in an expanded sample size using RT-qPCR. The present study showed significant elevation of the mRNA level of *FOXP1* in patients with ASD compared to that in the control group. Our finding suggests that increased *FOXP1* expression may be involved in the pathogenesis of ASD. However, the interpretation of the result should be cautious as there was a significant difference in the mean age between the two groups, which might be a confounder affecting *FOXP1* gene expression. We were not able to obtain blood from the children and young adolescents as the age-matched controls because of ethical considerations and patient protection, which is a limitation of this study. In addition, the findings from LCL can only be considered as indirect evidence reflecting what may happen in the brain.

The *FOXP1* gene encodes a member of the forkhead box transcription factor family that contains a DNA-binding domain and a protein-protein interaction domain. The *FOXP1* gene functions as a transcription repressor [39,41,42], and is widely expressed in the developing and mature brain. The gene has been suggested to be involved in the development and function of the brain [41,43]. In the literature, three subjects with mental retardation and significant language and speech deficits were detected to have heterozygous deletions overlapping the *FOXP1* gene [44]. Two mentally retarded individuals with autistic features were detected to have a *de novo* intergenic deletion and a *de novo* nonsense mutation in the *FOXP1* gene, respectively [45]. In an exome sequencing study of 20 sporadic ASD patients (simplex ASD), a *de novo* single-base insertion in *FOXP1* that introduces a frameshift and a premature stop codon was identified in a severely affected patient [46]. These data suggest that haploinsufficiency or hypomorphic mutations of *FOXP1* with reduced expression or deficient activity of *FOXP1* are associated with syndromic or non-syndromic ASD. However, these *FOXP1* mutations associated with ASD are rare; they may not apply to the pathogenesis of autism in general. In the present study, we found that increased *FOXP1* gene expression was associated with autism in general. Our finding may expand our understanding about the relationship of *FOXP1* with autism.

FOXP1 protein was known to interact with another subfamily member FOXP2 to form a heterodimer and co-expressed with FOXP2 in several brain regions, suggesting close functional collaboration between the two proteins [39,40]. Contactin-associated protein-like 2 (*CNTNAP2*), a neurexin family protein that functions as a neuronal adhesion molecule and receptor, was found to be a direct neural target bound by human FOXP2 protein [47]. Mutations of *FOXP2* and *CNTNAP2* were linked to speech and language disorders and ASD [36-38,48-51]. Taken together, these data indicate that interactions among *FOXP1*, *FOXP2*, and *CNTNAP2* genes may play an essential role underlying the pathogenesis of syndromic and non-syndromic ASD.

In a more recent study, forkhead box protein p1 was found to function as a transcriptional repressor of immune signaling in the mouse brain, and was involved in the pathophysiology of Huntington’s disease [52]. The study suggested that Foxp1-regulated pathways might be important mediators of neuronal-glial cell communication. Thus, the increased *FOXP1* gene expression in the LCL of ASD patients as found in this study may offer a new insight that dysregulated immune signaling in the brain contributes to the pathogenesis of ASD.

A previous study demonstrated that FOXP2 protein directly binds to intron 1 of the *CNTNAP2* gene and significantly downregulates the mRNA levels of *CNTNAP2*[47]. This interaction was further supported by the findings in a study of Foxp2 R553H knock-in mice [53], which showed a significant increase in the Cntnap2 mRNA level in the cerebellum of the transgenic animal [53]. Thus, we inferred that increased *FOXP1* gene expression may lead to increased expression of the *FOXP2* gene through a feedback mechanism, which may in turn reduce the gene expression of *CNTNAP2* in patients with ASD. We hypothesized that there might be significant differences in the gene expression of *FOXP2* and *CNTNAP2* in LCL between patients with ASD and control subjects. To address this issue, we attempted to measure the mRNA of *FOXP2* and *CNTNAP2* in LCL in our subjects using RT-qPCR. However, the mRNA levels of these two genes were too low to be detected in our system.

The present study may have several implications. First, as LCL is derived from the EBV transformation of lymphocytes of peripheral blood, it would be interesting to know whether there is also an increased *FOXP1* expression in the peripheral blood cells. If so, the increased *FOXP1* expression can be considered as a potential biomarker of ASD in future studies. Second, recent reports indicate that the expression of FOXP1 is subject to regulation by cis-acting SNPs, methylation, and microRNA [54-57], and thus, it would be interesting to explore the underlying mechanisms that may lead to increased expression of the *FOXP1* gene in ASD. Third, recent studies have demonstrated the feasibility of establishing induced neuronal cells from induced pluripotent stem cells (iPSC) [58] or directly from non-neuronal somatic cells [59]. Hence, it would be interesting to verify whether the increased expression of *FOXP1* in LCL of ASD patients can be observed in the induced neuronal cells. Furthermore, it would also be interesting to measure the expression levels of *FOXP2* and *CNTNAP2* in the induced neuronal cells that cannot be detected in LCL and investigate their interactions with *FOXP1*.

In a recent review of studies of genome-wide expression analysis in LCL or blood cells in ASD patients, Voineagu reported that not much of a convergent pathway was found in terms of dysregulated pathways from several studies he reviewed [60]. He provided several explanations for such discrepant findings, including differences in study designs, sample sizes, and the clinical heterogeneity of patients with ASD [60]. Nevertheless, given no overlap of specific pathways and genes among different gene expression studies of peripheral tissues in autism patients, he reported that an upregulation of several genes involved in immune response is common to several studies, which is compatible with the findings from several total gene expression studies of brain tissue in autism patients [60]. In the present study, when we carried out pathway analysis using three platforms, we found several immune-related pathways showing significant differences between the ASD patients and controls, including the RIG-I-like receptor signaling, the toll-like receptor signaling, the allograft rejection, the primary immunodeficiency, the cytokine-cytokine receptor interaction, the chemokine signaling, the intestinal immune network for IgA production, and the Jak-STAT signaling pathways. Our findings are compatible with several previous studies and support the notion that dysregulated immunological pathways may be involved in the pathogenesis of ASD [60]. In addition, we found several other pathways showing significant differences between the ASD patients and controls in three platforms, such as the long-term depression, the vascular smooth muscle contraction, the arrhythmogenic right ventricular cardiomyopathy, and the glycerophospholipid metabolism pathways. The detection of the long-term depression pathway is consistent with the finding from the study by Kong and colleagues [61]. Taken together, our findings in pathway analysis support the idea that autism is a complex genetic disorder.

## Conclusions

Our study suggests that comparative gene expression profiling analysis of LCL is a useful tool to discover novel genes associated with autism. In this study, we found a significant elevation of the gene transcript of *FOXP1* in patients with ASD, suggesting that increased *FOXP1* gene expression might be involved in the pathogenesis of ASD. Nevertheless, limitations of this study, such as the small sample size, a significant difference in the mean age between the two groups, and the non-brain tissue of the study, should be taken into consideration while assessing these findings.

## Abbreviations

ADI-R: Autism Diagnostic Interview-Revised; ARVC: Arrhythmogenic right ventricular cardiomyopathy; ASD: Autism spectrum disorders; DAVID: Database for Annotation Visualization and Integrated Discovery; EMA: A R package for Easy Microarray data Analysis; DSM-IV: Diagnostic and Statistical Manual of Mental Disorders, Fourth Edition; EBV: Epstein-Barr virus; GSEA: Gene set enrichment analysis; iPSC: Induced pluripotent stem cells; KEGG: Kyoto Encyclopedia of Genes and Genomes; LCL: Lymphoblastoid cell line; MINI: Mini International Neuropsychiatric Interview; PCR: Polymerase chain reaction; RMA: Robust Multi‒array Analysis; RT-qPCR: Real-time quantitative PCR; SNP: Single nucleotide polymorphism.

## Competing interests

The authors declare that they have no competing interests.

## Authors’ contributions

The contributions of the authors are as follows: SSG is the principle investigator of this project. SSG and CHC designed the study and wrote the protocol. SSG trained the clinical research team, supervised the research execution, and collected all the clinical data of ASD cases. SSG and YYW were responsible for the ADI-R training and interviews. SSG, YYW, WCT and CYS conducted clinical diagnosis and helped recruit the patients, and CHC screened for mental disorders in the controls. CHC and PHC conducted the gene expression analyses. WHC conducted the experimental work and analyzed the data. WHC drafted the manuscript. CHC and SSG critically revised the manuscript and all the authors read and approved the final manuscript.

## Supplementary Material

Additional file 1 List of the differentially expressed probe sets and genes identified in this study. Click here for file

Additional file 2 List of pathways showing differences between the Autistic Spectrum Disorder patients and controls using GSEA, DAVID, and EMA in this study. Click here for file
